# Gut-thyroid axis and celiac disease

**DOI:** 10.1530/EC-17-0021

**Published:** 2017-04-05

**Authors:** Aaron Lerner, Patricia Jeremias, Torsten Matthias

**Affiliations:** 1 B. Rappaport School of MedicineTechnion-Israel Institute of Technology, Haifa, Israel; 2 AESKU.KIPP InstituteWendelsheim, Germany

**Keywords:** intestine, thyroid, celiac disease, Hashimoto’s thyroiditis, autoimmunity

## Abstract

Autoimmune thyroiditis has an increased prevalence in patients with celiac disease and vice versa. The objective of the current review is to highlight the epidemiological, clinical, serological, pathological, pathophysiological, hormonal, genetic and immunological factors shared between the two entities. They might represent the two ends of the gut-thyroid axis where the cross-talks’ pathways are still unravelled. New observations are reviewed, highlighting some gut-thyroid interrelated pathways that potentially might lead to new therapeutic strategies.

## Introduction

### Hashimoto's thyroiditis

Hashimoto’s thyroiditis (HT) is one of the most common autoimmune endocrine diseases, characterized by an autoimmune-mediated destruction of the thyroid gland. Initially considered a rarity, HT has now become the most common autoimmune disease. Its prevalence is 0.8% when estimated from a review of published articles, 4.6% when estimated biochemically from the National Health and Nutrition Examination Survey and >5% when estimated from cytology of ultrasound-guided fine-needle aspirates of thyroid nodules. Similar to majority of the other autoimmune diseases, HT is age dependent and is more common in women (F:M ratio >10:1) (1). It is identified by anti-thyroglobulin (TG) and/or anti-thyroid peroxidase antibodies (TPO). The disease is a common cause of low thyroid hormones with a high thyroid-stimulating hormone (TSH) but some exhibit subclinical hypothyroidism. Interestingly, symptoms of HT and celiac disease (CD) often overlap (Table 1), and both share environmental, pathological, immunogenic, hormonal, serological and genetic aspects (2, 3, 4).

**Table 1:** Shared clinical features between celiac and autoimmune thyroid diseases.

**Symptom/sign**	**Celiac disease**	**Hashimoto’s thyroiditis**	**Graves’ disease**
Weight	Loss	Gain	Loss
Bowel movement	Diarrhea/constipation	Constipation	Diarrhea
Joint/bone pain	+/+	+/−, hypotonia	Muscle weakness
Fatigue/tiredness	+	+	+
Psychology	Depression, anxiety	Depression	Anxiety, nervousness, restlessness, attention and concentrating difficulties
Hair loss	+	+	+/− Alopecia
Infertility/missed periods	+/+	+/+	+/+
Miscarriage	+	+	+
Increased other autoimmune diseases	+	+	+

### Celiac disease

CD is an autoimmune inflammatory disorder of the small intestine, triggered by the ingestion of prolamins contained in wheat, barley or rye, in genetically susceptible individuals. Its incidence in the general population is 1–1.5%. It is gluten depended, thus early diagnosis and subsequent adherence to a gluten-free diet is highly recommended (5, 6).

Pathophysiologically, the enzyme tissue transglutamimnase (tTg) is the autoantigen. By posttranslational modification of the absorbed gliadin peptide, deamidating or crosslinking, those peptides are becoming immunogenic/toxic, resulting in mucosal inflammation and damage. Several well established serological markers are available for the diagnosis and follow up: anti-endomysium, anti-deamidated gliadin and anti-tTg autoantibodies. However, two novel ones, the anti-neo-epitope tTg and the anti-neo-epitope microbial Tg were recently described with good performances (7). The present review will concentrate on CD and HT, as an example of the gut-thyroid axis.

### Thyroid pathology in celiac disease

CD is associated with a number of autoimmune conditions, including HT. The prevalence of autoimmune thyroid disease (ATD) in patients with CD was suggested to be four times higher than that in the general population, though the range is very wide, spanning 1.2–30%. The range for HT in CD is narrower: 1.25–19% (8). Going vice versa, a Dutch study reported 21% of the patients with CD to have HT (8). As ATD is age depended, in the adult populations with CD the rate of hypothyroidism and/or HT is much higher than that in children, ranging between 12.9 and 30.5%. Symptoms of undiagnosed CD may be different in patients who also have thyroid disease. Recent studies have shown that it may be beneficial to screen ATD for CD as well (8, 9).

### Intestinal pathology in thyroid diseases

Only a single layer of epithelial cells separates the luminal contents from effector immune cells in the lamina propria and the internal milieu of the body. Breaching the epithelium can lead to pathological exposure of the highly immunoreactive sub-epithelium to the vast number of foreign antigens in the lumen, thus driving autoimmunogenesis (10). Increased interest on the thyroid-intestinal epithelium is supplied by the finding of 40% of patients with HT with lymphocytic colitis, higher intraepithelial lymphocyte counts, dilated tight junctions, and shorter and thicker microvilli. The gastrointestinal dysfunctions in thyroid disorders were most recently reviewed (11).

Taken together, a pathogenic role of the intestinal damage, including in CD, is suggested in the development of HT (12, 13). Several potential mechanisms were suggested for the gut mucosa and luminal ecosystems involvement in the thyroid autoimmunity: (i) Gut dysbiosis might disturb the finely tuned immune balance and break tolerance to self-antigens and non-pathogenic non-self-antigens, by posttranslational modification proteins, inducing autoimmunity; (ii) association of lipopolysaccharide-induced Toll-like receptor (TLR) activation with thyroiditis development or production of anti-thyroglobulin antibody in mice was suggested; (iii) induction of Th1 to Th2 shift, inhibition of Th17 differentiation and oral tolerance induction, by retinoic acid might activate tolerogenic immune responses in the gut; (iv) breaching tight junction integrity, resulting in the leaky gut barrier is a common shared pathway in autoimmunogenesis; (v) not less important is the transcriptomic, proteinomic and the metabolomics changes induced by the gut microbes, being direct messengers between the bugs and us. Changes in microbiota and short chain fatty acids production are clearly related to the pathogenesis of CD, but their role in thyroid autoimmunity induction or protection remains to be investigated (9, 10, 12, 13, 14, 15, 16).

### Shared autoantibodies

High positivity of anti-tTgs antibodies among subjects with ATD is well documented. It is recommended to have high clinical index of suspicion for CD in patients with AT. Higher percentage of anti-tTgs was described in several countries of Asia and Europe.

The opposite picture exists when anti-TPO/anti-TG antibodies are checked in CD. In a recent study, anti-thyroid antibodies became positive in 16.4% of the patients 2–3 years after the diagnosis of CD, once again alluding to the age dependence of the ATD.

### Shared genes

Common genetic predisposition has been proposed as a candidate explanation for the positive association between CD and ATDs. The putative Graves’ disease and HT susceptibility genes include both immune modifying genes and thyroid specific genes. The genes that predispose to endocrine autoimmune diseases, i.e. DR3–DQ2 and DR4–DQ8, are also the major genetic determinants of CD, which is the best understood human leukocyte antigen (HLA)-linked disease (17).

The immune-regulatory genes that predispose to autoimmune thyroid diseases (FOXP3, CD25, CD40, CTLA-4, the HLA genes, PTPN22) play critical parts in the development of an effective immune response including self-tolerance. More specifically, CD 40 is shared by Graves’ disease and inflammatory bowel disease (IBD), CTLA-4 is shared by Graves’ disease, HD, IBD and CD, HLA genes are shared by Graves’ disease, HT and CD, and finally PTPN22 is shared by CD, Graves’ disease, HT and IBD, thus enhancing the genetic cross-talks in the gut-thyroid axis (18).

### Shared immune pathways

Both diseases, share many immune-pathological pathways: ongoing chronic destructive inflammation and mononuclear infiltration of the target organs, predominance of T helper cell type 1 pattern response including the associated cytokines such as IL-18 and INF-γ. Notably, a shared plot was suggested between the two diseases and a new therapeutic strategy, namely, neutralizing antibodies against IL-18 and/or INF-γ were suggested as a fruitful option (2).

### Shared autoimmune diseases

Several autoimmune diseases are shared between CD and HT. CD is associated with numerous autoimmune diseases including HT. In a most recent review, multiple diseases were significantly associated with ATD, including CD (19).

### Microbiota/dysbiota/metabolome

The intestinal microbiota can be considered as an endocrine network. Although neglected by the endocrinologists for decades, gut microbiota microbiome represent an important endocrine organ that converts nutritional messages from the intestinal lumen into endocrine signals, impacting the metabolism of local as well as remote organs. Following are several examples of the nutrients-microbiotic-hormonal metabolic axes.

Several observations strengthen the endocrine impact of the microbiota. Hypo responsiveness toward epinephrine, norepinephrine and vasopressin during iodine deprivation, was observed in germ-free rodents. The intestinal microbes can regulate cholesterol, lipid and glucose metabolisms by their bile acid hydrolases capacity, resulting in hormonal release. One of the major metabolic products of the gut microbiome is short-chain fatty acids resulting in numerous luminal and systemic functions (15) affecting leptin, glucagon-like peptide 1, ghrelin and peptide YY and productions. The bug’s β-glucuronidases activate norepinephrine and dopamine. Their decarboxylation capacities induce γ-aminobutyric acid (GABA), tyramine and β-phenylethylamine productions. Finally, the plasma concentration of tryptophan is dependent on microbiome composition. Being an essential amino acid and precursor of serotonin, tryptophan impact enteric neurotransmitters balance (14).

Interestingly, microbiome can affect the cross-talks between the hypothalamic–pituitary–adrenal axis and behavior. In the opposite aspect, the endogenous hormones can modulate the bacterial proliferative capacity and pathogenicity. Dopamine, norepinephrine, nitric oxide and the inhibitory transmitter GABA are molecules originated from the luminal microbes that influence our endogenous endocrine network (referred to as ‘Microbial endocrinology’ (14)). The microbial intestinal-thyroiditis interrelations were not described in CD and IBD. Not surprising, probiotics were suggested as a potential therapy for IBD and HT. It can be summarized that in the gut lumen, this type of interrelationship between the bugs and us, is performed continually through various kinds of luminal messengers, part of the luminal metabolome and proteinome, which exert hormonal functions.

Multiple studies support the role of the intestinal commensal microbiota in autoimmunogenesis, including CD (10, 16). In contrast, the role of the intestinal indigenous microorganisms in HT has received little attention. Innate pattern recognition receptors such as TLRs stimulation and breached epithelial tight junction integrity were suggested to contribute to thyroid autoimmunity. Some literature data have suggested that dysbiosis could affect thyroid hormone synthesis and metabolism (20), and it had been suggested that gut bacteria could even deiodinate thyroid hormones, thus affecting serum levels of these hormones (21).

The gut is the first and the widest area of bacteria access, with the highest concentration of T-cells in the human body and trained to react to microorganisms. Interestingly, all the environmental factors involved in the pathogenesis of CD, HT and Graves’ disease can alter the balance within the microorganisms located in the gut, and influence the immune system, in particular the proportions of regulatory Treg and inflammatory TH17 cells (15, 22).

However, the link between the intestine and HT was scarcely investigated, resulting in indirect and weak evidence for such a link, at least, till today (12).

## Similarities and dissimilarities between Celiac disease and autoimmune thyroiditis

The two diseases share multiple similarities and dissimilarities. Following are Tables 1 and 2, summarizing the clinical pictures shared or unshared between CD, HT and Graves’ disease (Table 1) and comparison of various features between CD and HT (Table 2).

**Table 2:** Comparison of various features between celiac and Hashimoto thyroiditis.

	**Celiac disease**	**Hashimoto thyroiditis**
Incidence	1–1.5%, increases	5%, increases
Gender predominance	Female	Female
Geoepidemiology	Increasing incidence	Increasing incidence
Environmental factors	Gluten, microbial mTg, infection, stress, formula feeding, increased diversity of dysbiota	Infection, diet, iodine, medications, smoking
Associated infections	Enterovirus, EBV, CMV, HBV, HCV, rotavirus *Bacteroides* species, *Campylobacter jejuni*, *pneumococcus*, tuberculosis and *Helicobacter pylori*	EBV, Yersinia enterocolitica, Helicobacter pylori, HCV, CMV, Borrelia burgdorferi
Dysbiota	Decreased diversity	?
HLA predisposition	DQ-2, DQ-8	HLA-DRβ1-Arg74, DQ-2
Autoantibodies	tTg, DGP, EMA, neo-epitope tTg, neo-epitope mTg	Anti-thyroid peroxidase, anti-thyroglobulin
Autoantigen	tTg	Thyroid peroxidase, thyroglobulin
Potential inducer enzyme (PTMP)	tTg, mTg deamidation/cross-linking	TTg
Adaptive/innate immunity	+++	+++
Target/associated organs	Small bowel/joint, bone, endocrine, heart, lung, liver, kidney, skin, nerves, etc.	Thyroid
Therapy	Gluten free diet	Symptomatic, thyroid replacement therapy

CMV, cytomegalo virus; DGP, diamidated gliadin peptide; EBV, Epshtein Bar virus; EMA, endomysial antibodies; HBV, hepatitis B virus; HCV, hepatitis C virus; mTg, microbial transglutaminase; tTg, tissue transglutaminase.

## Gluten and autoimmune thyroiditis

Literature does not support the use of gluten-free diet (GFD) in the routine treatment of ATD. The possible role of gluten in the induction of the anti-thyroid antibodies as well as, in few cases, the consequent organ dysfunction was suggested. Interestingly, thyroid disease was 3-fold higher in CD than that in controls. In most patients who strictly followed a 1-year GFD, there was a normalization of subclinical hypothyroidism, suggesting that in distinct cases, gluten withdrawal may single-handedly reverse the abnormality. It seems that facing thyroid-associated orbitopathy, CD is the only autoimmune disease where complaints and autoantibodies to tTg usually resolve on a GFD. At least one antibody was positive in 10 of 19 untreated celiacs but only in five of 25 gluten-restricted patients. Once again it shows that gluten withdrawal may change thyroid autoimmunity, mainly when associated with CD.

## Gut-thyroid interrelated pathways

### Molecular mimicry

An unexpected cross-reaction of the antibodies with autologous components may occur. This process is in fact at the basis of the progression of autoimmune diseases and is called molecular mimicry. In the gut, inflammatory pathologies that are related to dysbiosis associated with various factors, such as genetic factors and food, cause alterations of the immune system characterized in IBD and CD (9, 10, 14). Those enteric eco-events induce systemic inflammatory responses, leading to the systemic manifestations of IBD and/or CD, affecting remote organs including the thyroid (10, 14).

### Gut luminal eco-events that might drive gut-thyroid axes

#### Dysbiosis

The balanced interaction between the host and microbes has been shaped during the long co-evolutionary process. In dysbiotic conditions, this balance is compromised and results in abnormal interaction between the host and microbiota (10). In contrast, the place of the altered microbiome, mainly decrease diversity, in autoimmunity induction was well described (9, 10, 14). Adding the substantial involvement of the microbiome in IBD and CD development and the potential place in HT evolvement, dysbiosis is suggested as a major player in intestinal and thyroidal autoimmunity interaction (12, 13).

#### Posttranslational modification of proteins (PTMP)

PTMP contribute substantially to the adaptability and bacterial cell cycle regulation. Their enzymatic apparatus is capable to transform naïve/self- or non-self-peptides to autoimmunogenic ones (10). In this regard, CD is a prototype of PTMP by the endogenous tTg and potentially by the microbial Tg (9, 10, 14). Further support for the gut-thyroid axis in the recent report of anti-tTg autoantibodies binding to thyroid follicles and extracellular matrix opens a new window for tTg-induced PTMP involvement in the thyroidal autoimmunity (23).

#### Leaky gut

Numerous autoimmune diseases expose increased intestinal permeability (9). This phenomenon was described in CD (9, 14), IBD and thyroidal dysfunction (24). Finally, as microbes are a major breacher of the tight junction integrity and as dysbiosis is crucial in autoimmunogenesis, the leaky gut is assumed to enhance thyroid dysfunction.

### tTg and thyroid tissue dysfunction

tTg antibodies were described to bind to thyroid follicles and extracellular matrix in patients with CD, thus reinforcing the gut-thyroid relationship (13, 23). More so, the anti-tTg titers correlate with TPO antibody titers (23). These findings suggest that celiac-associated autoantibodies could contribute to the development of thyroid dysfunction in CD.

### Mucosal stress: the heat shock protein theory

Heat shock proteins (HSP) are stress proteins that have a role in protection of cellsagainst stress. Increased HSP expression was noticed in the intestine and serological anti-HSP autoimmune response was detected in patients with CD and IBD (25). Hsp60 levels are increased in HT and are immunolocalized in the thyroid tissue. Anti-TG and anti-TPO antibodies cross-react with Hsp60 (26). Those relations need more thorough investigations.

## Conclusions

**Figure 1:**
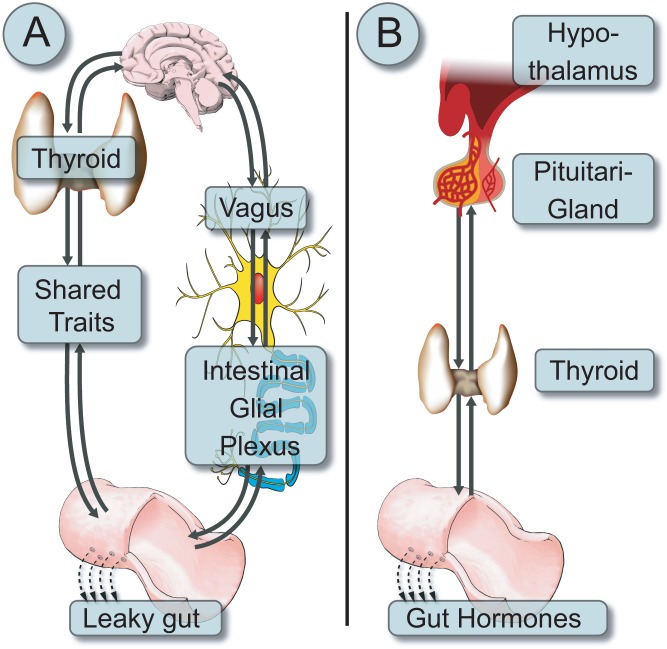
A schematic presentation of (A) the bidirectional neuronal pathways connecting the thyroid through the vagal nerve to the intestinal neuronal plexus, finally inducing leaky gut. Parallel, multiple gut-thyroid shared traits (epidemiology, autoantibodies, genes, immune pathways and autoimmune diseases) influence the gut-thyroid axis; (B) the hormonal bidirectional cross-talks between the hypothalamus-pituitary-thyroid-gut hormones axes.

Despite 104 years of Hashimoto’s thyroiditis, and 128 years of CD discovery, both are still intriguing diseases. Multiple epidemiological, clinical, serological, pathological, pathophysiological, genetic, hormonal and immunological aspects are shared between the two (Fig. 1A and B). Increased prevalence of CD-associated antibodies is well described in HT and many recommend screening HT populations for celiac autoimmunity. The routine screening of CD for thyroid autoimmunity is less established (27). Understanding the cross-talks between the gut eco-system events, the intestinal hormonal repertoire and the thyroid gland (28) might open new therapeutic strategies to combat those diseases.

## Declaration of interest

The authors declare that there is no conflict of interest that could be perceived as prejudicing the impartiality of this review.
